# A diagnostic odyssey of herpes zoster mimicking pacemaker pocket infection

**DOI:** 10.1093/ehjcr/ytae052

**Published:** 2024-01-29

**Authors:** Kostopoulou Anna, Cheilas Vasileios, Martinos Antonios, Michalis Efremidis

**Affiliations:** Onassis Cardiac Surgery Center, Electrophysiology Lab, Syngrou 356, 17674 Kallithea, Athens, Greece; Onassis Cardiac Surgery Center, Electrophysiology Lab, Syngrou 356, 17674 Kallithea, Athens, Greece; Onassis Cardiac Surgery Center, Electrophysiology Lab, Syngrou 356, 17674 Kallithea, Athens, Greece; Onassis Cardiac Surgery Center, Electrophysiology Lab, Syngrou 356, 17674 Kallithea, Athens, Greece

A 73-year-old post-transcather aortic valve implantantion and pacemaker-implanted woman presented with localized redness and pain in the pacemaker pocket region (*Figure 1A*). Due to initial concerns of an isolated pocket infection, empirical vancomycin administration was initiated after obtaining blood cultures.^1^ Despite the general recommendation of a 2–3-day washout period in stable patients to enhance the precision of microbiological diagnosis, we opted for an earlier initiation in consideration of the patient’s frailty and the presence of a prosthetic valve. This decision was tailored to the specific circumstances of the patient to mitigate the risk of further complications. A transthoracic echocardiogram was conducted to identify lead vegetations and assess valvular involvement. Additionally, a transoesophageal echocardiogram was initially scheduled, but the characteristic progression of the skin lesion prompted a reconsideration of this decision since during the next 2 days, a pale multiforme erythematous lesion with vesicles (*Figure 1B* and *C*) unfolded, unmasking the herpes zoster antagonist.^2^ In this point, is worth mentioning, that the patient did not have any history of herpes zoster before. Intravenous acyclovir precipitated rapid improvement within a week (*Figure 1D* and *E*), while lingering pain and sensitivity persisted for over a month despite complete erythema healing in two weeks (*Figure 1F*).^3^ This case highlights the need for identification of distinctive features of rashes suggestive of a pocket infection, consideration for tailored antibiotic initiation, the contemplation of withholding antibiotics in stable patients with concurrent presumed pacemaker pocket infection and confirmed zoster inflammation, and strict adherence to established guidelines for herpes zoster vaccination to prevent similar clinical complexities.

**Figure 1:**
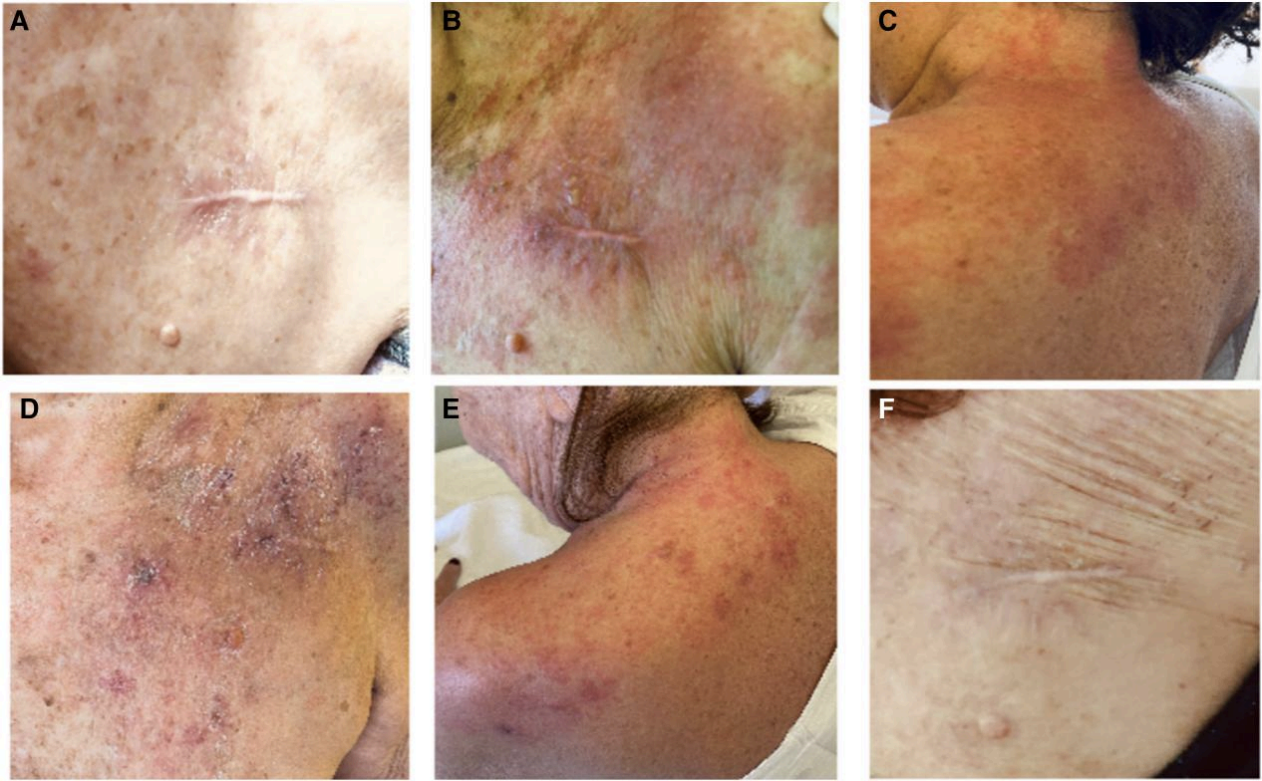
Diagnostic odyssey of herpes zoster mimicking pacemaker pocket infection.

**Consent:** The authors affirm obtaining written consent from the patient for the submission and publication of this case report, inclusive of images, adhering to COPE guidelines.

**Funding:** This work received no external financial support. The authors conducted this investigation independently, driven by their commitment to advancing medical knowledge.

## Data Availability

The data underlying this article are available in the article.
